# Clear cell sarcoma of the kidney in a 62-year-old patient presenting with generalized pruritus

**DOI:** 10.1186/s12885-019-6212-1

**Published:** 2019-11-01

**Authors:** Yuxi Zhang, Jun Li, Yan Wang

**Affiliations:** 1 Department of Urology, The First Hospital of China Medical University, No. 155 Nanjing North Street, Shenyang, 110001 China; 2 Department of Pathology, The First Hospital and College of Basic Medical Sciences, China Medical University, No. 155 Nanjing North Street, Shenyang, 110001 China

**Keywords:** Clear cell sarcoma of the kidney, Pruritus, Immunohistochemistry, Vimentin, BCL-6 corepressor, Chemotherapy, Doxorubicin

## Abstract

**Background:**

Clear cell sarcoma of the kidney (CCSK) is the second most common renal tumor in children following Wilms’ tumor. CCSK is extremely rare in adults, with only 25 adult cases reported in the medical literature.

**Case presentation:**

We reported a 62-year-old man with a right renal mass presenting only with generalized pruritus who underwent radical right nephrectomy. With immunostaining, tumor cells were positive for expressed vimentin, neural cell adhesion molecule (NCAM, CD56), and Ki-67 and focally positive for p53, CD10 and Bcl-2. The histopathological diagnosis was CCSK. Two weeks after the operation, the generalized pruritus ended. One month after the operation, the patient started treatment with a regimen combining doxorubicin, vincristine, cyclophosphamide, and etoposide. At the 20-month follow-up visit, there was no evidence of local recurrence or metastases.

**Conclusions:**

In a patient presenting with generalized pruritus, further evaluation for an underlying malignancy should be considered. It is difficult to distinguish CCSK from undifferentiated renal neoplasms. Immunohistochemistry could help to make exact histopathological diagnoses. The BCL-6 corepressor (BCOR) gene could play a significant role in CCSK tumorigenesis and be a good marker for CCSK diagnosis. Surgery with combination chemotherapy and radiation therapy could be used to treat CCSK in older patients.

## Background

Clear cell sarcoma of the kidney (CCSK) is the second most common renal tumor in children following Wilms’ tumor. The average age at diagnosis of CCSK is 3 years old. The tumor is prone to metastasize to bone, brain, and soft tissue [1]. The symptoms of CCSK are abdominal or flank mass, abdominal pain, hematuria, asthenia, anorexia, weight loss, low-grade fever, nausea, vomiting, constipation, anemia, Stauffer syndrome, and high blood pressure [2]. We present a case of a 62-year-old man with CCSK presenting only with generalized pruritus. We discuss the histopathologic diagnosis, genetic studies of CCSK and the efficacy of surgery with combination chemotherapy in elderly individuals (older than 60 years).

## Case presentation

**Fig. 1:**
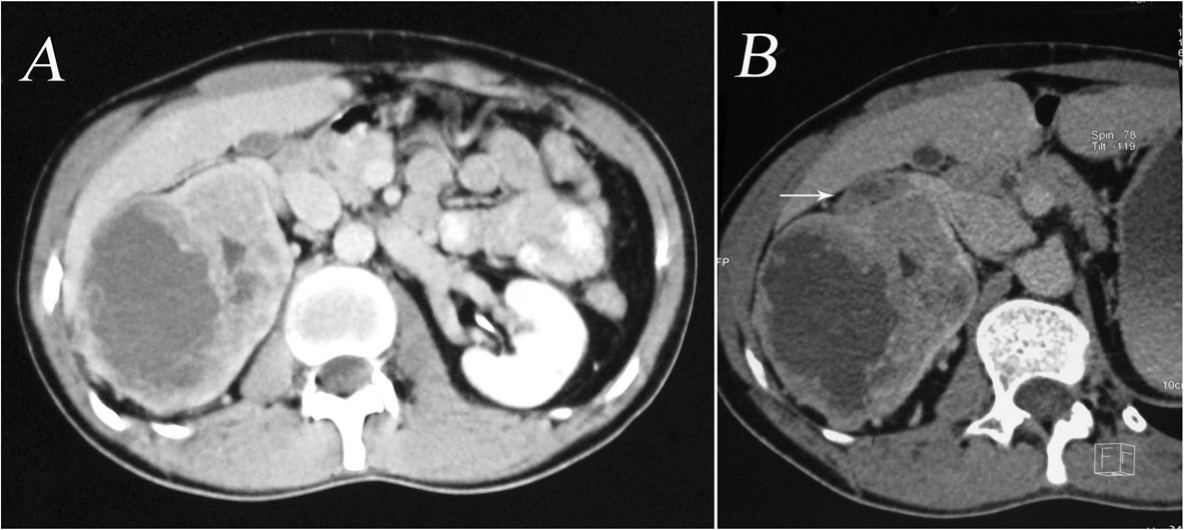
**a**. CT scan showing an 8.0-cm right-side heterogeneous renal mass infiltrating the renal calices. **b**. CTA showing a right-side renal tumor with tumor thrombus in the right renal vein (white arrow)

A 62-year-old man with a 45-day history of generalized pruritus since February 2017 presented to the medical department. He had been diagnosed in another hospital with senile pruritus and had been treated by antihistamines and emollient cream for 20 days. The itch was not completely relieved, and he came to seek further treatment. He did not smoke cigarettes or drink alcoholic beverages. He had no notable medical history except for hypertension. On physical examination, there were no notable findings except for slight scratch marks. Blood tests showed normal erythrocyte, leukocyte and platelet counts, and urinalysis and chest radiography showed nothing of note. There was no evidence of renal or thyroid dysfunction. Laboratory studies revealed normal liver chemistry. The erythrocyte sedimentation rate (ESR) was 35 mm/h. A color duplex sonography of the abdomen revealed a large hypervascular, low-level echo, heterogeneous right renal mass. An intravenous pyelogram revealed irregularity of the margin of the right renal calices. Computed tomography (CT) scan of the abdomen and the pelvis showed a heterogeneous mass originating from the upper pole of the right kidney and infiltrating to renal calices (Fig. 1a). Helical CT angiography (CTA) demonstrated a right renal tumor with a tumor thrombus in the right renal vein that did not extend into the inferior vena cava (IVC) (Fig. 1b). Further evaluation, including a bone scan, did not demonstrate any evidence of metastases.

A right radical nephrectomy with a complete regional intraperitoneal lymphadenectomy with an anterior subcostal incision was performed in April 2017. No intraoperative or postoperative complications developed.

**Fig. 2:**
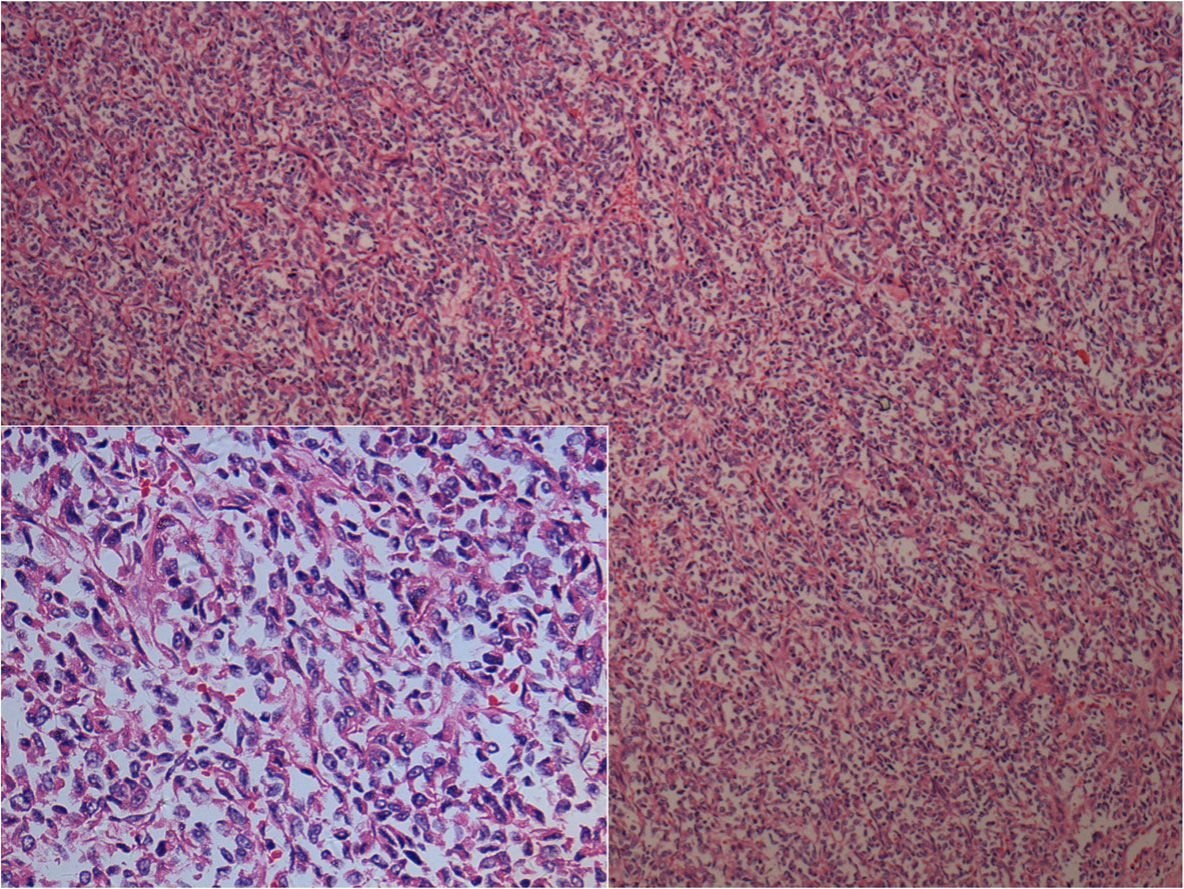
Histopathologic examination showing the classic pattern of CCSK. The tumor consists of nests and cords of cells separated by fine, arborizing fibrovascular septa. The nuclei were optically clear with fine chromatin, were round to oval in shape, and lacked prominent nucleoli and mitotic structures (H&E100×, insert H&E 400×)

**Fig. 3:**
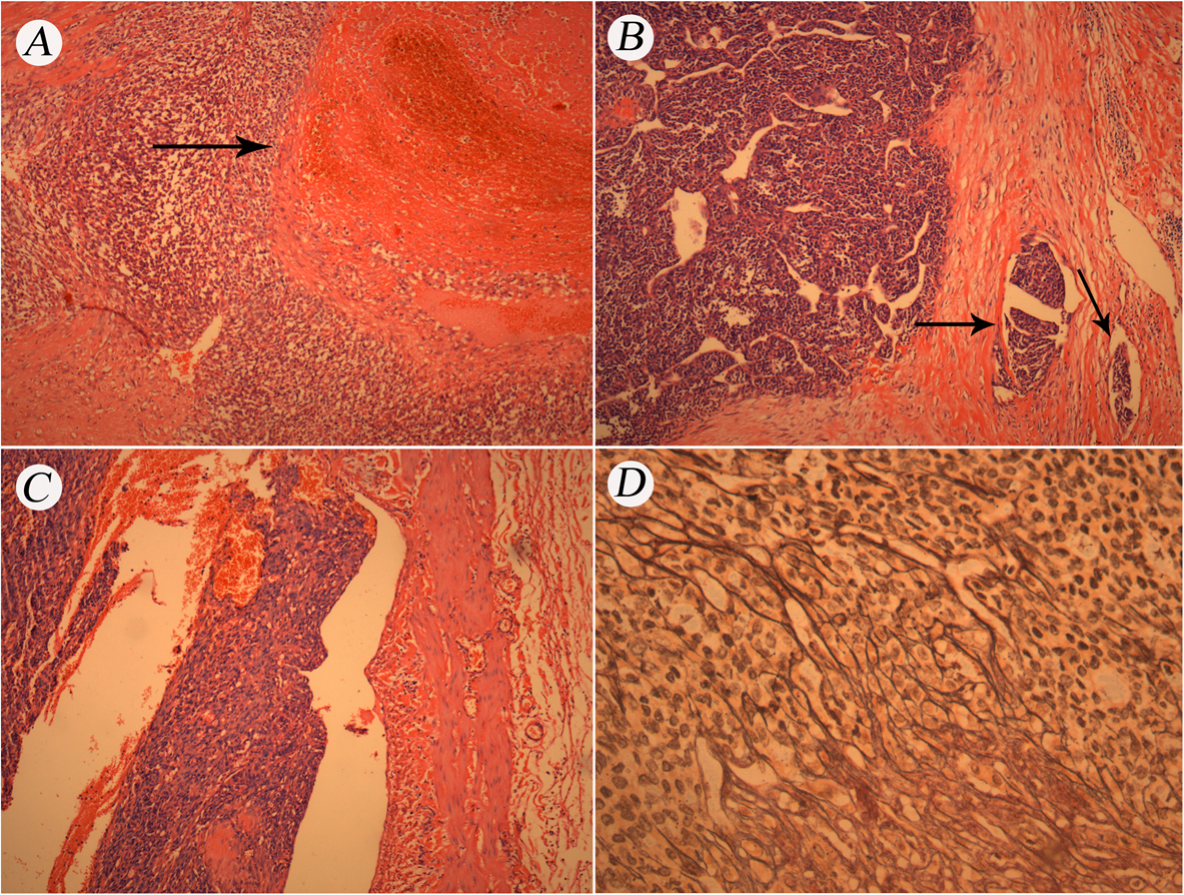
**a**. Microscopic examination showing necrosis in the tumor (black arrow, H&E 100×). **b**. Microscopic examination showing micro-tumor thrombi in small vessels of the tumor (black arrow, H&E 100×). **c**. Microscopic examination showing a tumor thrombus in the renal vein (H&E 100×). **d**. Silver stain clearly demonstrated reticular fibers often outlining individual tumor cells (400×)

**Fig. 4:**
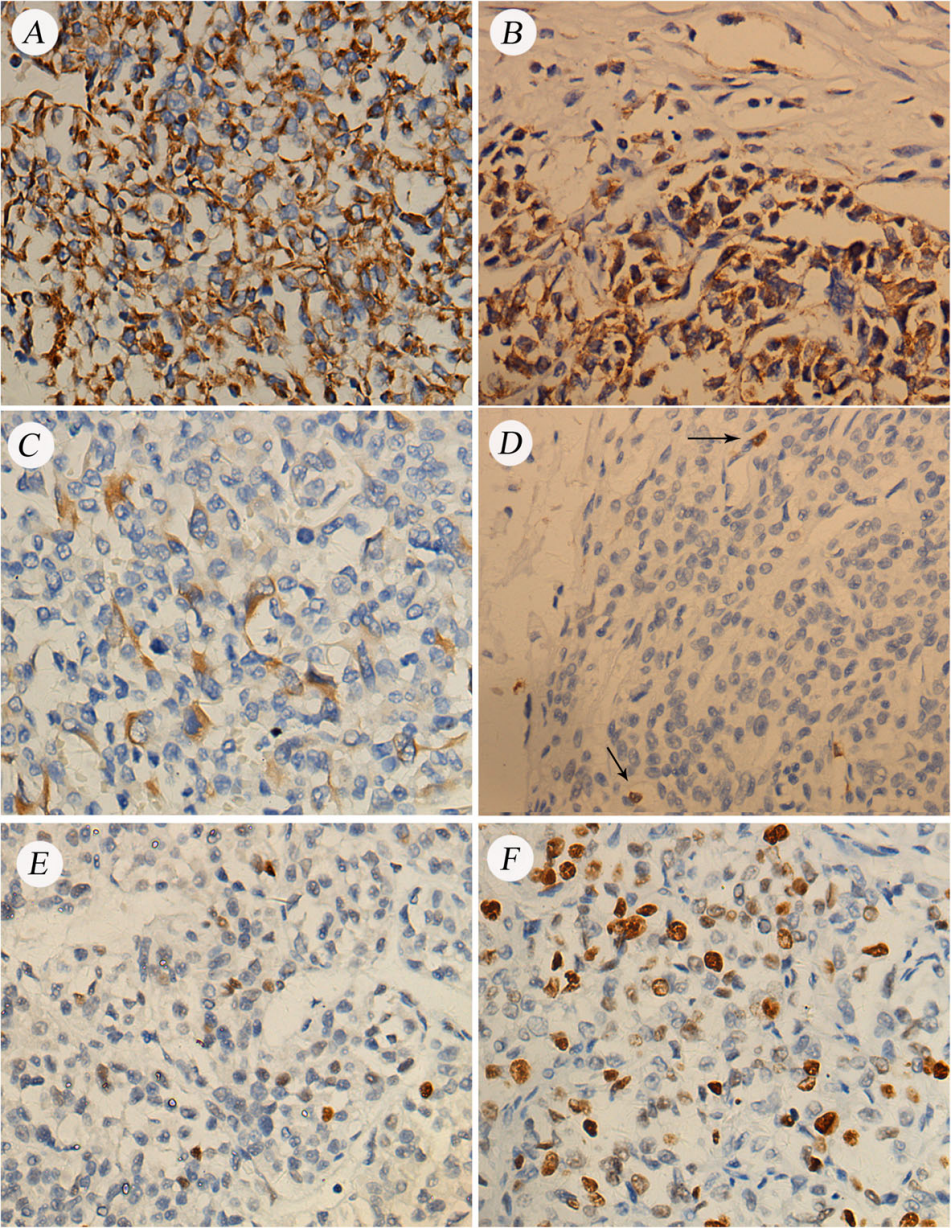
Immunostaining of the tumor with positive reactivity for (**a**) vimentin and (**b**) CD56, as well as focally positive reactivity for (**c**) Bcl-2, (**d**) CD10 (black arrow), (**e**) p53, and (**f**) Ki-67. (400×)

**Fig. 5:**
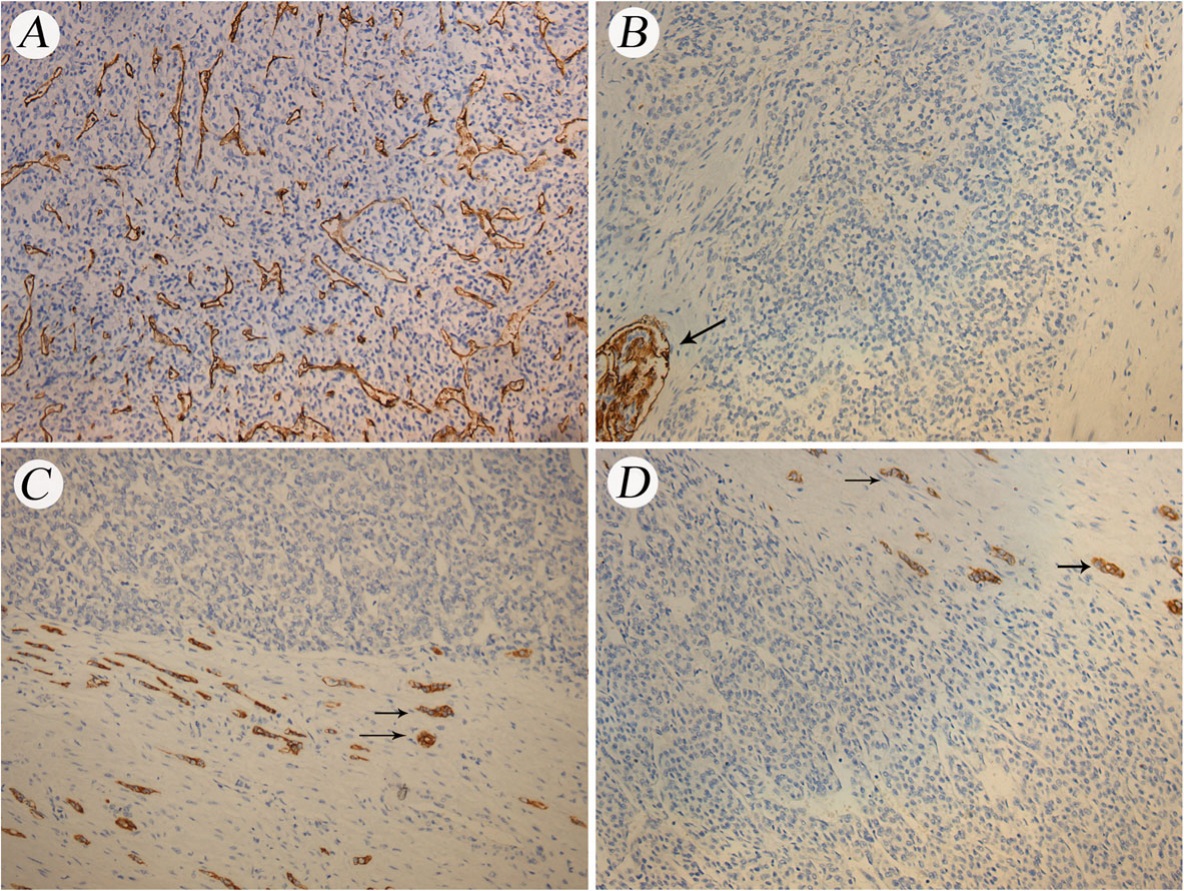
Immunostaining of the tumor negative for CD34, RCC-Ma, EMA, and CAM5.2 (200×). **a**. CD34 showing reactivity with the prominent vascular network in the tumor. **b**. RCC-Ma showing reactivity with the luminal surface of Bowman’s capsule adjoining the tumor (black arrow). **c**. EMA showing reactivity with the renal tubules adjoining the tumor (black arrow). **d**. CAM5.2 showing reactivity with the renal tubules adjoining the tumor (black arrow)

Gross examination showed a 9 × 8 × 8 cm daffodil yellow and madder red tumor with foci of necrosis. No positive surgical margins were discovered. There was a 2 cm yellowish-red soft tumor thrombus in the right renal vein without infiltration into the vessel wall. The tumor thrombus did not extend into the IVC but did infiltrate into the renal calices. No lymph node metastases were found. We obtained 12 random specimens from the tumor for histopathologic examination except for the tumor thrombus. Microscopy revealed that the mass consisted of nests and cords of cells separated by fine, arborizing fibrovascular septa. The collagenous material intermingled among tumor cells, which were cuboidal or polygonal in shape. The nuclei were optically clear with fine chromatin and round to oval in shape, lacked prominent nucleoli, and exhibited infrequent mitosis (Fig. 2). There was necrosis in the tumor (Fig. 3a). Figure 3b shows microtumor thrombi in small vessels of the tumor. The histopathologic characteristics of the tumor thrombus in the renal vein were the same as those of the tumor in the kidney (Fig. 3c). Silver staining clearly demonstrated reticular fibers often outlining individual tumor cells (Fig. 3d), suggesting that the tumor was a sarcoma but not a carcinoma. Immunostaining was performed to make an exact histopathologic diagnosis. With immunostaining, tumor cells were positive for expressed vimentin, neural cell adhesion molecule (NCAM, CD56), and Ki-67 and focally positive for p53, Bcl-2 and CD10 (Fig. 4). Immunostaining for alpha B-crystallin, alpha-smooth muscle actin, CAM5.2, CD34, chromogranin (Chr), Cytokeratin7, M2A oncofetal antigen (D2–40), Desmin, epithelial membrane antigen (EMA), hemopoietic cell kinase (HCK), neuron specific enolase (NSE), P63, renal cell carcinoma marker (RCC-Ma), S100, Wilms’ tumor 1 (WT1), CD57 and CD15 were negative. We carefully checked an internal positive control for the negative staining. We found a prominent vascular network with positive reactivity for CD34 in the tumor (Fig. 5a). RCC-Ma was positive in the luminal surface of Bowman’s capsule adjoining the tumor (Fig. 5b, black arrow). EMA and CAM5.2 were expressed in renal tubules adjoining the tumor (Fig. 5c, d, black arrow). These data showed that the histopathologic diagnosis of the mass was CCSK. The pathological diagnosis was made by consultation of three pathologists in our hospital. The finalized pathologic stage was T3aN0M0 based on the American Joint Committee on Cancer (AJCC) definition and accepted as stage II according to the updated National Wilms Tumor Study 5 (NWTS-5) definition.

Two weeks after the operation, the generalized pruritus ended. One month after the operation, the patient started treatment with a regimen combining doxorubicin (DOX), vincristine (VCR), cyclophosphamide, and etoposide. Twenty months after the operation, CT of the abdomen and pelvis revealed no evidence of local recurrence in the right kidney. Other examinations revealed no evidence of distant metastases. The patient is still alive and without clinical evidence of progression. The patient can complete daily life activities, occasionally feeling tired and experiencing numbness at the incision site.

## Discussion

We report a case of CCSK in an aged patient presenting with generalized pruritus. CCSK was first described in 1970 by Kidd [3] and is prone to metastasize to bone, brain, and soft tissue. The symptoms of CCSK are abdominal or flank mass, pain in the abdomen, blood in the urine and high blood pressure [1, 2]. To the best of our knowledge, our present case is the first documented case of CCSK presenting with generalized pruritus. There are two kinds of pruritus associated with malignancy: itch induced by local reaction to malignancy and paraneoplastic itch (PI) [4]. PI is defined as itch that may occur early during tumor formation or even precede clinical evidence of the malignancy. It is not caused by neoplastic mass invasion or compression and subsides after removal of the tumor [5]. PI is also defined as a systemic (not local) reaction to the presence of a tumor or a hematological malignancy, neither of which is induced by the local presence of cancer cells or by tumor therapy. It usually disappears with remission of the tumor and can return with its relapse [6]. We thought that the pruritus in our patient was PI. The epidemiological data of PI are limited. Kilic reported that generalized pruritus was present in 13% of 700 solid tumor and hematological cancer patients [7]. Other studies investigated the underlying etiology of idiopathic generalized pruritus and found that malignancy is a cause in less than 10% of patients [8–10]. Lymphoma and leukemia were the most common malignancies. It can also be part of a rare paraneoplastic syndrome resulting from solid tumors, including those in the lung, colon, breast, stomach and prostate [11]. The mechanism of pruritus caused by cancer is unclear [6]. In a patient presenting with generalized itching, further evaluation for an underlying malignancy should be considered. Its recognition may lead to early diagnosis and improved outcome.

CCSK is the second most common renal tumor in children following Wilms’ tumor and forms approximately 5% of pediatric renal tumors. The average age at the time of diagnosis of CCSK is 3 years old [1]. CCSK is extremely rare in adults. We reviewed the literature in PubMed and found that there were only 25 cases reported from 1989 to 2018 [12–30]. In the 25 adult cases, excluding our case, the mean age was 34.5 years (from 16 to 70 years). There were only two cases of CCSK in patients older than 60 years [26, 30]. Here, we report the third elderly case of CCSK. CCSK tends to occur in children, with a male preponderance (male/female ratio 2/1) [1]. For the 25 adult cases, the ratio between males and females was 17:8, which was similar to that in children.

In our case, there was a tumor thrombus in the right renal vein without infiltration into the vessel wall. In the 25 adult cases, excluding our case, there were three cases with IVC tumor thrombus [17, 24, 27] and two cases with tumor thrombus extending into the right atrium [21, 23]. The mean age was 38.6 years (from 22 to 55 years). The ratio between males and females was 4:1, and the incidence rate of tumor thrombus was 20% (5/25). We report on the oldest CCSK patient with a tumor thrombus.

The pathological diagnosis of CCSK is very difficult. Kidd firstly reported that CCSK was a distinct clinicopathologic entity in 1970 [3]. Then the distinctive histopathologic features of CCSK were reported in 1978 [31–33]. It has been emphasized that CCSK showed tremendous morphologic diversity, ranging from epithelioid to spindle cell patterns [34]. CCSK has several histological pattern variants, including myxoid, sclerosing, cellular, epithelioid (trabecular or acinar type), palisading, spindle cell, storiform, and anaplastic. The typical gross features of CCSK are large size, mucoid texture, foci of necrosis, and prominent cyst formation. The tumors are most commonly described as tan– grey, soft, and mucoid on cut section. There are common discrete foci of necrosis and hemorrhage in the tumors. The classical light microscopic features of CCSK are defined as nests or cords of cells separated by regularly spaced, arborizing fibrovascular septa. Classical CCSK is composed of nests and cords of cells with scant cytoplasm and high nuclear-cytoplasmic ratios. The nuclei are characterized by a fine chromatin pattern, and mitotic structures are generally rarely identified. The tumor has a prominent vascular network and abundant collagenous extracellular matrix material, and isolated nephrons are entrapped by the tumor [1, 2]. The renal tumors showing the typical macroscopic and microscopic pathological findings of CCSK should be further identified by immunohistochemistry (see below). The pathologic features of CCSK in aged patients are unclear, but we found they were similar to those in pediatric patients in our case. We suggested that.

CCSK is rare in elderly patients. To exclude renal cell carcinoma (RCC), we examined the histological variation within the large tumor. Twelve specimens were obtained from the tumor randomly and examined carefully. Microscopic examinations showed the same pathologic features in the twelve specimens. There were no other neoplastic components in the tumor. We used silver staining to further examine the twelve specimens. As shown in Fig. 3d, silver impregnation clearly demonstrated reticular fibers often outlining individual tumor cells, which showed that the tumor might be a sarcoma but not a carcinoma. RCCs sometimes exhibit a sarcomatoid appearance known as sarcomatoid renal cell carcinoma [35]. Some studies have demonstrated epithelial features even in the sarcomatoid component of this tumor [35, 36]. The correct diagnosis of undifferentiated RCCs is difficult. The undifferentiated RCCs can be misdiagnosed as renal sarcomas. The accurate pathological diagnosis of renal tumor is very important because the undifferentiated RCCs do not benefit from any adjuvant therapy at the moment, whereas renal sarcoma might be a candidate to specific adjuvant therapies. RCCs frequently react with antibodies to brush border antigens and low-molecular-weight cytokeratins such as CK8, CK18, CK19, AE1, and CAM 5.2 [37–39]. RCC-Ma is a monoclonal antibody against a normal renal proximal tubule antigen whose expression is relatively specific for major RCCs [37, 40]. The majority of RCCs react positively for EMA [41]. However, these markers are negative in CCSK [1]. In our case, the results of immunostaining showed that CAM5.2, CK7, EMA and RCC-Ma were negative in the tumor, indicating that this tumor was not an RCC.

CCSK is frequently confused with other undifferentiated renal tumors, including blastema-predominant Wilms’ tumor, primitive neuroectodermal tumor (PNET), cellular congenital mesoblastic nephroma (CMN), and malignant rhabdoid tumor of kidney (MRTK). We suggest that immunohistochemistry in CCSK should be used to distinguish CCSK from undifferentiated renal neoplasms. A complete immunohistochemical panel including vimentin, cytokeratin, WT-1, Desmin, and markers for neural differentiation and myogenic origin are needed for the diagnosis of CCSK. Almost all other immunohistochemical markers such as CD34, S100, desmin, CD99, cytokeratin, and EMA are uniformly negative in CCSK, while vimentin and Bcl-2 are typically reactive [1, 22, 42]. Satoh et al. reported that diffuse and strong positivity of CD56 is characteristic of CCSK. They also reported focal positivity of CD10 and negativity of CD57, NK1, CD15, EMA, CA15–3 and WT-1 [43]. As shown in Table 1, we believe that negativity of all these markers is more important than positivity for vimentin, CD56, and CD10. Vimentin is diffusely positive in MRTK cells, while cytokeratins and EMA are variably positive. MRTK is less commonly focally positive for other markers such as S100, NSE, synaptophysin, and CD57 [44]. PNET is mainly positive for CD99, NSE, vimentin, S100, and synaptophysin in up to 60% of cases. CD57 is variably positive in PNET [45]. However, these proteins, except vimentin, are negative in CCSK. WT1, Desmin, NSE, and cytokeratin cocktail CK22 are negative in CCSK but positive in blastema-predominant Wilms′ tumor [46–48]. Cellular CMN displays cytoplasmic immunoreactivity for vimentin, desmin, muscle actin (HHF-35), and alpha-smooth muscle actin [49]. In our study, the tumor cells were positive for vimentin, CD56, and Ki-67 and focally positive for p53, CD10 and Bcl-2. However, other markers were negative. We used this information to make the diagnosis of CCSK.

**Table 1:** Differential positive immunohistochemical markers in CCSK and other undifferentiated renal neoplasm [1, 22, 42–49]

Undifferentiated renal neoplasm	Positive markers
CCSK	vimentin, CD56
Blastema-predominant Wilms’ tumor	vimentin, NSE, desmin, WT1, cytokeratin cocktail CK22
PNET	CD99, NSE, vimentin, S100, synaptophysin, CD57
MRTK	Vimentin, cytokeratin, EMA, S100, NSE, synaptophysin, CD57
Cellular CMN	vimentin, desmin, muscle actin (HHF-35), alpha Smooth muscle Actin

*CCSK* clear cell sarcoma of the kidney, *PNET* primitive neuroectodermal tumor, *MRTK* malignant rhabdoid tumor of kidney, *CMN* congenital mesoblastic nephroma, *EMA* epithelial membrane antigen, *NSE* neurone specific enolas

The tumorigenesis of CCSK is unclear. Karlsson considered that CCSK might originate from embryonic mesenchymal progenitor cells [50]. The BCL-6 corepressor (BCOR) gene was found to regulate mesenchymal stem cell function by epigenetic mechanisms [51]. Ueno-Yokohata found 100% internal tandem duplications (ITDs) in exon 15 of the BCOR gene in 20 cases of CCSK [52]. Other studies also found BCOR ITDs in CCSK [53–55]. Argani considered BCOR to be a sensitive and specific marker for pediatric CCSK [56]. BCOR ITDs could also be detected in circulating tumor DNA in CCSK preoperative cases [57]. Thus, BCOR could play a significant role in CCSK tumorigenesis and be a good marker for CCSK diagnosis. Gene fusions, including YWHAE-NUTM2B/E and IRX2-TERT, were discovered in a minority subgroup of CCSKs [58, 59]. However, the role of YWHAE-NUTM2B/E and IRX2-TERT fusion was not clear. It is interesting that the presence of the BCOR ITDs is mutually exclusive with the presence of the YWHAE-NUTM2B/E fusion in CCSKs [55]. Gooskens analyzed changes in chromosome copy number, mutations, rearrangements, global gene expression and global DNA methylation in CCSKs. They identified no recurrent segmental chromosomal copy number changes or somatic variants. They found a single case with the YWHAE-NUTM2 fusion in 13 cases of CCSK. The promoter hypermethylation and low expression of the tumor suppressor gene TCF21 were identified in all CCSKs except the case with a YWHAE-NUTM2 fusion. The hypermethylation of TCF21, a transcription factor known to be active early in renal development, might lie within the pathogenic pathway of most CCSKs [60]. Hence, the exact molecular pathogenesis of CCSKs remains poorly understood, especially that of adult CCSKs. The status of BCOR ITDs, YWHAE-NUTM2B/E fusion and hypermethylation of TCF21 in adult CCSKs is not clear. Unfortunately, the changes in BCOR ITDs, YWHAE-NUTM2B/E fusion and hypermethylation of TCF21 were not examined in our case. Future studies are needed to reveal the tumorigenesis of adult CCSKs.

The survival of patients with CCSK has increased from only 20 to 70% [1, 61]. Kusumakumary et al. reported that the relapse rate of CCSK was approximately 65% and the mortality rate was 48%. Half of the deaths occurred within the first two years. The prognosis for low-grade or early stage CCSK has improved with the addition of DOX to chemotherapy regimens [62]. Important predictors of improved survival are low stage, young age at diagnosis, treatment with DOX, and absence of tumor necrosis [1].

It is suggested that surgery, radiotherapy and chemotherapy should be used to treat CCSK together or separately. NWTS-3 showed that the addition of DOX to the combination of VCR, dactinomycin, and radiation therapy resulted in improved disease-free survival for patients with CCSK (Table 2) [61]. In the NWTS-4 study, compared with patients treated for 6 months, patients treated with VCR, DOX, and dactinomycin for 15 months had improved relapse-free survival (RFS) (Table 2) [63]. The NWTS-5 study revealed that children with stage I to IV CCSK were treated with a new chemotherapeutic regimen combining VCR, DOX, cyclophosphamide, and etoposide in an attempt to further improve the survival of these high-risk groups. All patients received radiation therapy to the tumor bed. With this treatment, the 5-year event-free survival (EFS) was approximately 79% (95%CI, 69 to 86%), and the overall survival (OS) was approximately 89% (95% CI, 80 to 94%) with a median follow-up of 4.6 years after diagnosis. The 5-year EFS for stage I is 100%, stage II is 87%, stage III is 74%, and stage IV is 36% since diagnosis. The 5-year OS for stage I is 100%, stage II is 97%, stage III is 87%, and stage IV is 45% since diagnosis (Table 2) [64]. The latest results observed on NWTS-5 showed that 5-year EFS and OS were 79% (95% CI: 71–88%) and 90% (95% CI: 84–96%) with a median follow-up of 9.7 years after diagnosis by incorporating cyclophosphamide and etoposide into treatment (Table 2) [65]. The best choice of treatment for CCSK in adults is still unknown. Some studies have reported that surgery with combination chemotherapy decreased the probability of recurrence [23, 26]. We treated the patient with surgery followed by combination chemotherapy of VCR, DOX, cyclophosphamide, and etoposide. The patient did not receive radiation therapy. There was no evidence of local recurrence or metastases for approximately 20 months. We thought that surgery with combination chemotherapy and radiation therapy might be a good choice of treatment for CCSK in older patients.

**Table 2:** Therapeutic outcome of CCSK from NWTS studies

Report	Year	Study	n	RFS	EFS	OS
Green [61]	1994	NWTS 1–2 NWTS 3	66 73	25–63.5%(6y) 58.2–64.4%(6y)	NA NA	25–71.9(6y) 60.8–71.3%(6y)
Seibel [63]	2004	NWST 4	86	65.2–87.8%(5y) 60.6–87.8%(8y)	NA NA	87.5–95.5%(5y) 85.9–87.5%(8y)
Seibel [64]	2004	NWST 5	110	NA	79%(5y)	89%(5y)
Seibel [65]	2019	NWST 4 NWST 5	68 108	NA NA	72%(5y) 79%(5y)	87%(5y) 90%(5y)

*CCSK* clear cell sarcoma of the kidney, *NWTS* National Wilms Tumor Study, *RFS* relapse-free survival, *EFS* event free survival, *OS* overall survival, *NA* not available

## Conclusions

In conclusion, further evaluation for malignancy should be considered in patients who present with generalized pruritus. The pathologic features and treatment of CCSK in older patients were similar to that in pediatric patients. It is difficult to distinguish CCSK from undifferentiated adult renal neoplasms, although immunohistochemistry could help to make histopathological diagnoses. Surgery with combination chemotherapy and radiation therapy could be used to treat CCSK in these patients. The tumorigenesis of adult CCSKs should be characterized in the future.

## Data Availability

All data generated or analyzed during this study are included in this published article [and its supplementary information files].

## Abbreviations

AJCC: American Joint Committee on Cancer
BCOR: BCL-6 corepressor
CCSK: clear cell sarcoma of the kidney
Chr: chromogranin
CMN: congenital mesoblastic nephroma
CT: Computed tomography
CTA: CT angiography
DOX: doxorubicin
EFS: event-free survival;
EMA: epithelial membrane antigen
ESR: erythrocyte sedimentation rate
HCK: hemopoietic cell kinase
ITD: internal tandem duplication
IVC: inferior vena cava
MRTK: malignant rhabdoid tumor of kidney
NCAM, CD56: neural cell adhesion molecule
NSE: neuron specific enolase
NWTS: National Wilms Tumor Study
OS: overall survival
PI: paraneoplastic itch
PNET: primitive neuroectodermal tumor
RCC: renal cell carcinoma
RCC-Ma: renal cell carcinoma marker
RFS: relapse-free survival
VCR: vincristine
WT1: wilm’s tumor 1
